# Early development and substrate twine selection for the cultivation of *Sargassum muticum* (Yendo) Fensholt under laboratory conditions

**DOI:** 10.1007/s10811-018-1459-5

**Published:** 2018-04-23

**Authors:** Hau Nhu Le, Adam D. Hughes, Philip D. Kerrison

**Affiliations:** 1 Scottish Association for Marine Science (SAMS), Scottish Marine Institute, Dunbeg, Argyll 1QA UK; 2 Nha Trang Institute of Technology and Application (NITRA), Vietnam Academy of Science and Technology (VAST), 2 Hung Vuong Street, Nha Trang, Vietnam

**Keywords:** *Sargassum muticum*, Phaeophyta, Hatchery, Nutrient, Cultivation, GeO_2_, Twine

## Abstract

The phaeophyte macroalgae *Sargassum muticum* is under investigation as a cultivation crop within its native range in SE Asia, alongside other members of the *Sargassum* genus. During the critical hatchery phase, germlings are grown to ≥ several millimeters ready for outplanting. By optimising the growth medium and twine substrate used for the germling attachment, hatcheries can become more efficient and cost-effective. An 8-week replicated laboratory experiment investigated these factors. It found that adding 0.125 mL L^−1^ of saturated germanium dioxide during the first week increased mean germling size by 23% (*p* < 0.005), whereas additional nutrients in the form of F/2 medium made no difference (*p* > 0.05). Six twine substrates were also tested: jute, cotton, polyamide/cotton, polyester, polyvinyl alcohol and polypropylene. *Sargassum muticum* grew similarly well on all, although attachment success during the first week was highest on the rougher natural fibres, particularly jute. A negative density-dependent effect of germling density on growth was seen across all materials, with the highest growth seen on the materials with the lowest germling density. Jute is recommended as a highly suitable substrate for hatchery cultivation in this species, although the initial density should be carefully controlled to prevent intraspecific competition.

## Introduction

*Sargassum muticum* is native to coastal China, Japan and Korea, where it can form extensive monospecific or mixed sub-littoral canopies (Yoshida 1983). These canopies provide numerous ecosystem services such as coastal protection, primary production, nutrient cycling and spawning, nursery and feeding areas for species of commercial importance (Coston-Clements et al. 1991; Tsukidate 1992; Al-Hafedh et al. 2015). *Sargassum* sp. stands are harvested for food, traditional medicine, feed, fertiliser and chemical products (Sohn 1993; Hong et al. 2007), which have led to widespread overexploitation. This problem is exacerbated by other anthropogenic interferences such as pollution and land reclamation (Mata et al. 2016).

To ensure a continued supply and provide seed stock for the restoration of natural beds, cultivation methods are under development for many Sargassaceae species (Titlyanov et al. 2012; Redmond et al. 2014; Yoon et al. 2014). *Sargassum muticum* is currently cultivated within its native range in SE Asia (Cao et al. 2008; Liu et al. 2013), along with a number of its relatives including *Sargassum fusiforme* (Pang et al. 2005), *Sargassum fulvellum* (Hwang et al. 2006), *Sargassum horneri* (Pang et al. 2009), *Sargassum naozhouense* (Xie et al. 2013), *Sargassum thunbergii* (Zhao et al. 2008) and *Sargassum vachellianum* (Chai et al. 2014). Germlings are initially attached to twines or fabrics that may be grown within the hatchery for 3–5 months before they are outplanted into the sea (Redmond et al. 2014). Optimising the juvenile growth and development within the hatchery will reduce costs and maximise efficiency.

*Sargassum muticum* is considered to be one of the world’s most successful invasive seaweeds (Denny 1988). Due to its wide physicochemical tolerance for growth and reproduction combined with varied dispersal methods, it is an opportunistic space-grabber (Fletcher and Fletcher 1975; Kerrison and Le 2016). These characteristics have allowed it to spread rapidly around the Atlantic coast of North America and Europe following accidental introductions (Critchley et al. 1983). It now ranges from the cold waters of southern Alaska and Norway to the warmer waters of Mexican Pacific and the Mediterranean (Cheang et al. 2010). In some locations, the invasion has displaced native species of macroalgae or seagrass by forming dense stands which overgrow and outcompeting the native flora (Fletcher and Fletcher 1975; Denny 1988; Callow et al. 2000; Bitton et al. 2006), whilst in other cases, little or no impact is seen (Dexter et al. 1975).

It has previously been determined that egg release and germling production are maximised at 20 °C and 50–100 μmol photons m^−2^ s^−1^ (Kerrison and Le 2016). Furthermore, a 3-min treatment of 0.5% potassium iodide with 0.38% sodium hypochlorite can be used to remove grazing epibionts from either adults or 6-week-old germlings, with little physiological impact (Kerrison et al. 2016a). In other members of the Sargassaceae and kelp such as *Saccharina latissima*, there is ongoing research to optimise the hatchery phase through manipulation of the nutrient growth medium (Kerrison et al. 2016b) and the addition of the diatom inhibiting compound (GeO_2_) germanium dioxide (Shea and Chopin 2007). The use of GeO_2_ is considered equally applicable to the cultivation of *S. muticum*, and so, it has been used previously during *Sargassum* sp. cultivation experiments (Fletcher and Fletcher 1975; Huggett et al. 2009; Heydt et al. 2012), although the benefit has not be validated or the dosage optimised.

It is known that *S. muticum* requires a hard surface for attachment of the holdfast. This includes anything from shells or pebbles up to bedrock or man-made structures (Fletcher and Fletcher 1975; Norton 1977; Critchley et al. 1983). Settling germlings are coated in extensive mucilage which is reported to adhere to whatever substrate they encounter, with the attachment tenacity increasing over time as the adhesive is secreted by the developing rhizoid mass (Norton 1980; Norton and Fetter 1981). A rugose surface is most favourable, allowing greatest retention of young germlings when exposed to water motion (Malm et al. 2003). The species also has the unusual ability to colonise soft substrata; following the initial settlement, the developing adult is partially buried allowing to remain secured, despite buoyancy provided by internal gas bladders (Strong et al. 2006).

Various strings are used for the cultivation of *Sargassum* spp., including mixes of polyamide (PA), cotton and polypropylene (PP) (Hwang et al. 2006; Zhao et al. 2008; Pang et al. 2009; Xie et al. 2013). However, the authors are not aware of any study comparing the suitability of different synthetic or natural twine. The utilisation of the best twine may allow the optimal hatchery development of *Sargassum* spp. germlings.

The first aim of this study was to determine whether the hatchery development of juvenile *S. muticum* could be optimised by the addition of nutrients or dosing with GeO_2_. The second aim was to compare the attachment and growth of *S. muticum* on six commercially available twines, to determine which was best for the cultivation of this genus.

## Materials and methods

### Algal materials

**Fig. 1:**
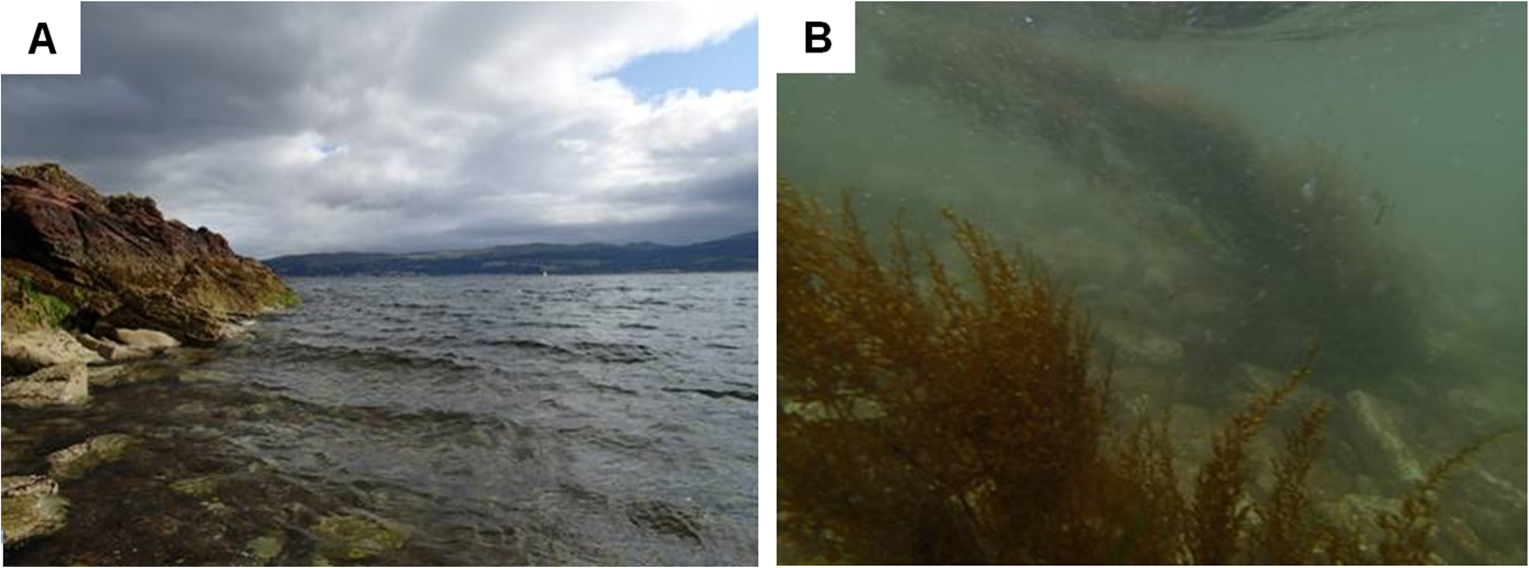
Invasive *Sargassum muticum* adults were collected from Great Cumbrae, UK (**a**) in August 2014, where they form subtidal canopies (**b**)

Fertile specimens of *Sargasum muticum* were collected in August 2014, at 0.5–1.0 m below chart datum from Great Cumbrae Island, Western Scotland (Fig. 1; 55° 45.211 N, 004° 54.070 W), based on observations made in previous reports (Harries et al. 2007). Cool boxes of natural seawater were used to transport these to the Scottish Association for Marine Science (SAMS), Oban, within 3 h. Selected thalli were healthy and yellowish-brown in appearance with swollen receptacles which had no obvious shedding. These were gentled cleaned of obvious epiphytic macroalgae and animals using a camel-bristled brush then weighted and submerged within outdoor 70-L aerated tanks of sand-filtered seawater under natural condition (13–16 °C, 100–200 μmol photons m^−2^ s^−1^).

### Liberation and collection of germlings

Within 2 weeks, of incubation in outdoor tanks, receptacles had matured and begun to shed in the typical acropetal sequence (Kerrison and Le 2016). Receptacle-bearing branches from ~ 1.5 kg were excised, rinsed and then incubated within two indoor 70-L tanks of sand-filtered seawater (20–22 °C, 50–80 μmol photons m^−2^ s^−1^) for 36 h. Preliminary trials showed that after 36 h under these conditions ~ 70% of all eggs had detached from the parent branches and sink vertically, adhering to the available substrate as described in Kerrison and Le (2016). Gentle brushing was used to detach mature eggs and germlings from the bottom of the tanks and from the receptacles. These were then collected using a 125-μm filter and washed and re-filtered several times in Tyndallised seawater (Kawachi and Noël 2005). The size and number of zygotes were then measured under a light microscope (Zeiss-Axioskop, Germany) and photographed with a connected camera (Canon EOS1100D, Taiwan).

### Germling settlement

**Fig. 2:**
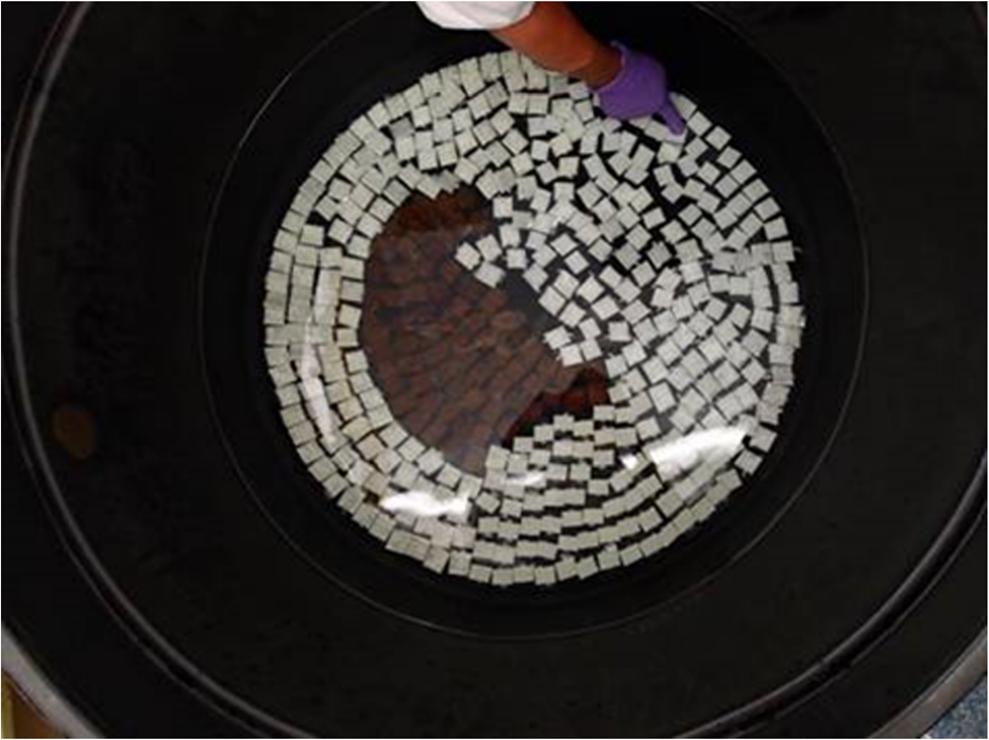
Zygote settlement was conducted simultaneously on all materials tested. Glass slides wrapped with 24 × 24 mm area of settlement twine were placed on the bottom of a large tank. The non-motile germlings were then allowed to settle

**Fig. 3:**
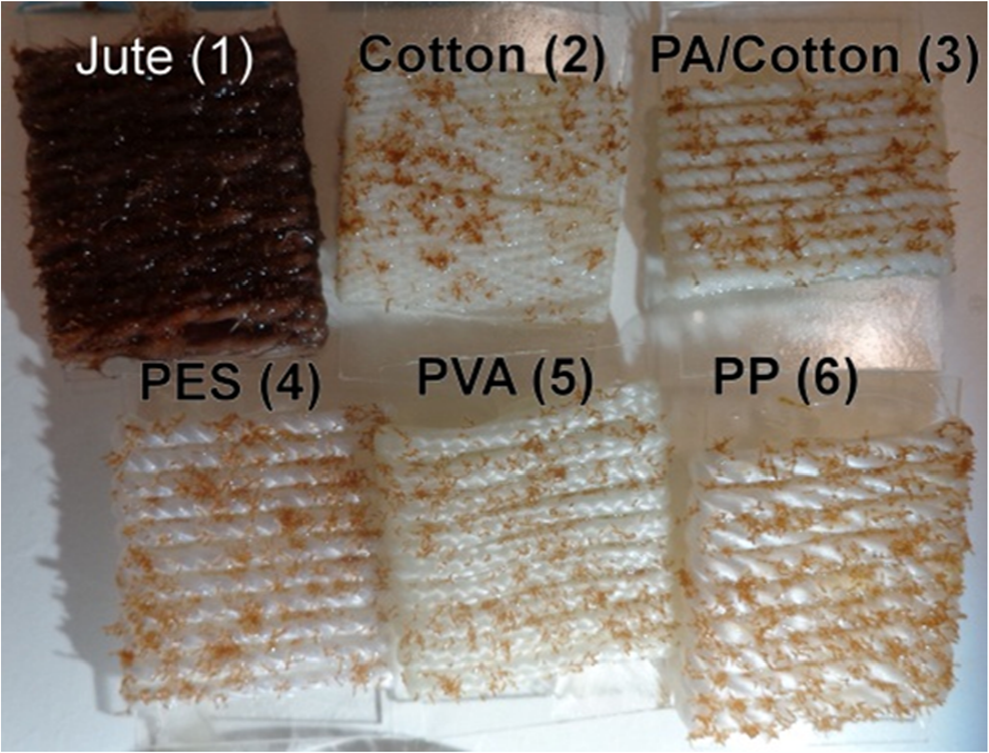
Six twines were tested for their suitability for the cultivation of *S. muticum*. For each, a ~ 24 × 24 mm area was presented for their settlement. Shown are twines at week 6 with attached germlings

A tank with a bottom area of 0.5 m^2^ was filled with 10 L of Tyndallised seawater enriched with F/2 medium (Guillard 1975) without silicate (F/2-Si) and 0.125 mg L^−1^ GeO_2_ to prevent diatom growth (Markham and Hagmeier 1982; Kerrison et al. 2016b). Glass slides (20 × 38 cm), bearing a ~ 24 × 24-mm area of wrapped settlement twine, were distributed on the bottom of the tank (Fig. 2). The twines were jute, cotton, PA/cotton, polyester (PES), polyvinyl alcohol (PVA) and PP (Fig. 3). These were soaked overnight in 5% Decon90 (Decon Laboratories Ltd, UK), rinsed thoroughly with distilled water and dried at 40 °C. The zygote suspension was then sprayed evenly over the water surface and 2 h was allowed for the non-motile zygotes to settle to the bottom. This resulted in a settlement density of 240 ± 14 zygotes cm^−2^ (mean ± S.D., *n* = 10) and a germination rate of 92%.

### Incubation experiment

**Fig. 4:**
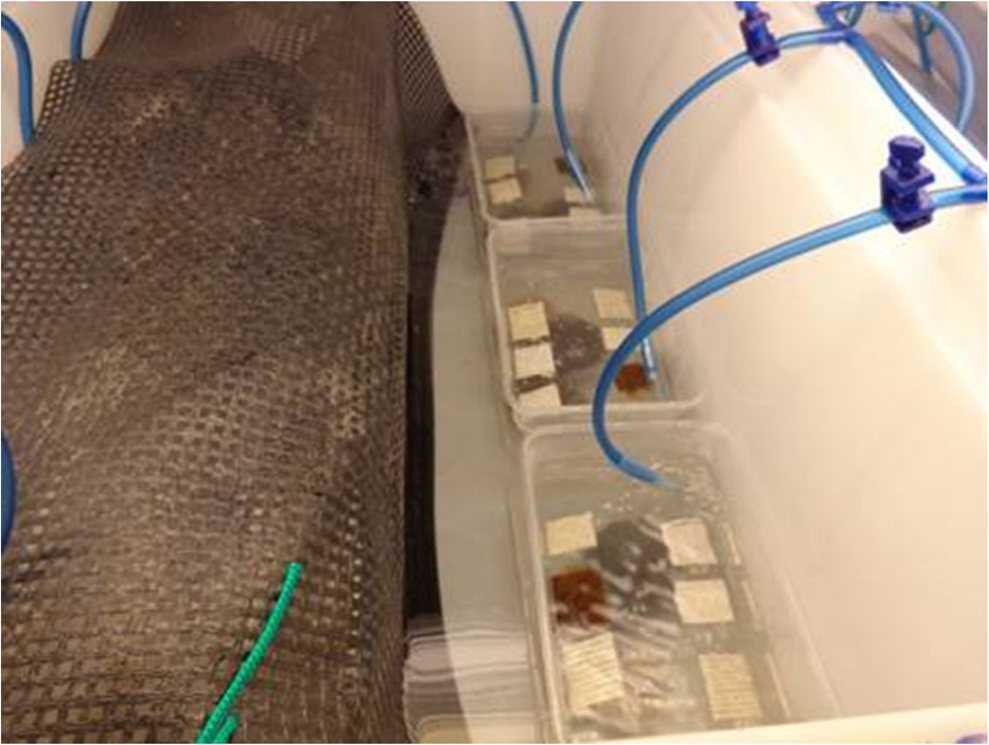
The twines were cultivated in triplicate tubs (400 mL) at a set temperature within a water bath. Each tub was bubbled gently

Settlement slides were then transferred into tubs containing 400 mL of seawater medium with transparent lids (Fig. 4). The tubs were then incubated in triplicate at 25 ± 0.5 °C and under 20–150 μmol photons m^−2^ s^−1^; 12:12 h light:dark cycle. This irradiance variation has a little significant impact on *S. muticum* growth (Kerrison PD, Le HN, Hughes AD (unpublished results)). The temperature was maintained using controllers (WH7016, WILLHI, China) linked to a thermocouple and 100–200 W water heaters. Lighting was provided by overhead fluorescent lights (cool white 40 W, Philips). Each tub was bubbled gently with lab air during the first week, with stronger aeration provided for the remaining period. The settlement slides were cultured for 8 weeks, with weekly media refreshment.

The Tyndallised seawater used was supplemented to test the effect of additional nutrients and GeO_2_. The conditions tested were unenriched seawater, enriched with F/2-Si, and enriched with F/2-Si and GeO_2_ for the first week and enriched with F/2-Si and GeO_2_ throughout the whole experiment.

At the start of the experiment and each week, a photograph of each slide was analysed in ImageJ software v1.46r (National Institutes of Health, USA). The germling density was determined, survival rate was calculated and the length of 10 germlings was recorded.

### Statistical testing

Minitab v.15 (Minitab Inc) and Excel 2010 (Microsoft) were used for statistical calculations. One-way (AN) and two-way analysis of variance (2wAN), where significant, were followed by post-hoc Tukey’s tests for comparisons between conditions. Parametric assumptions were evaluated and data transformed as necessary to fit these assumptions. Linear regression (LR) was used when a linear relationship was predicted.

## Results

### Morphological variation of germlings

**Fig. 5:**
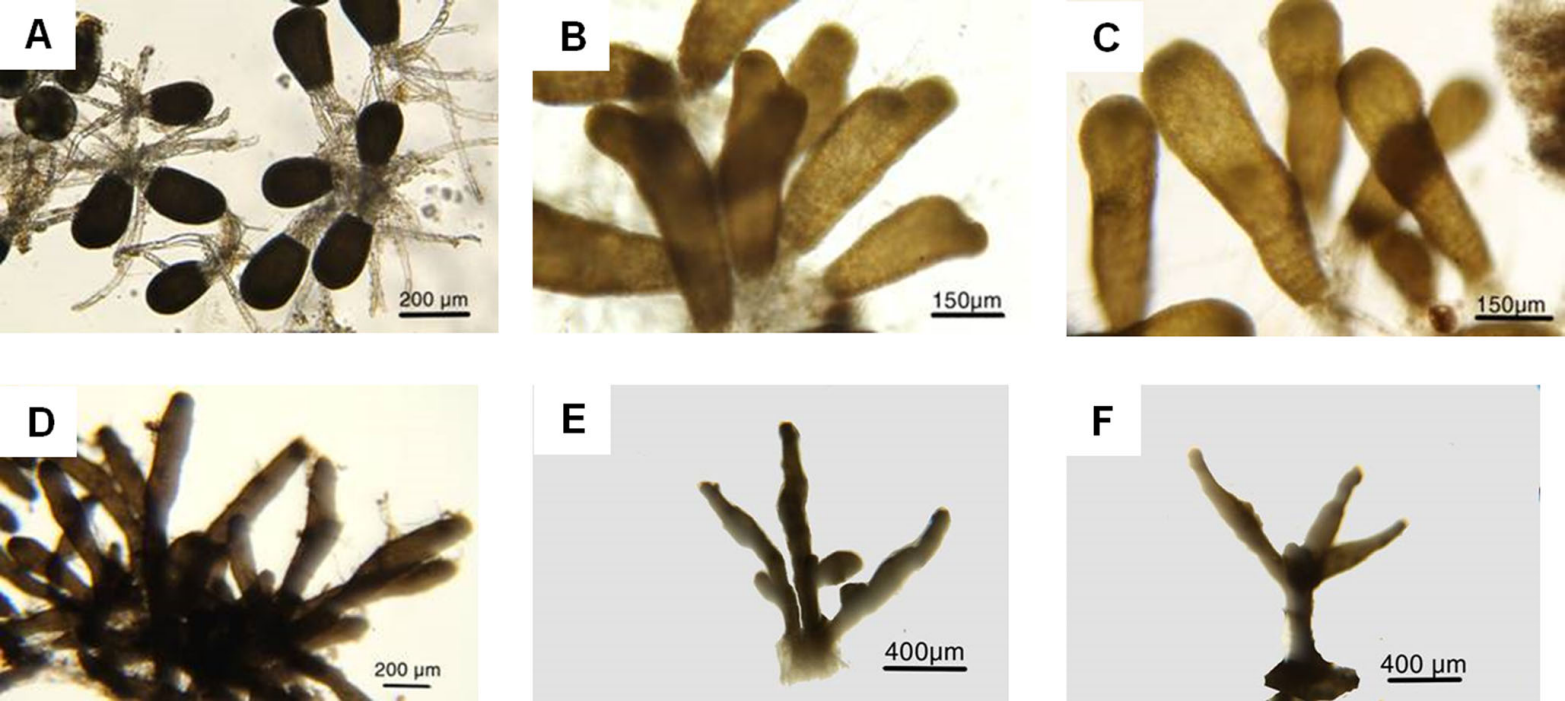
The development of *Sargassum muticum* germlings over 8 weeks. **a** On the 3rd day following zygote release from the adults, eight transparent rhizoids were clearly visible. **b** After 1 week, **c** 2 weeks and **d** 4 weeks, germlings were growing rapidly, with further basal rhizoids visible. **e** At 6 weeks, the germlings were beginning to branch into separate fronds. **f** These were clearly visible at week 8, along with a clear holdfast

At liberation, germlings had a diameter of 125 ± 5 μm and length of 195 ± 5 μm (Fig. 5). Following settlement, the germlings develop rapidly, forming an erect shoot axis and the basal production of eight transparent tubular rhizoids. After 3 days, the rhizoids had a mean length of 314 ± 25 μm, attaching the germlings firmly to the settlement slides. After 1 week further basal rhizoids were produced, and the germlings had a length of 320–650 μm and 1–4 apical hairs 900–1200 μm composed of long columnar cells. In week 5, germling attained a length of about 1 mm. By week 6, when 1.2–1.3-mm long, a small basal bud was produced which developed into the leaf-like lamina. At week 8, more laminae were produced, whilst numerous secondary rhizoids coalesced to form the holdfast.

### Germling growth and density over 8 weeks

**Fig. 6:**
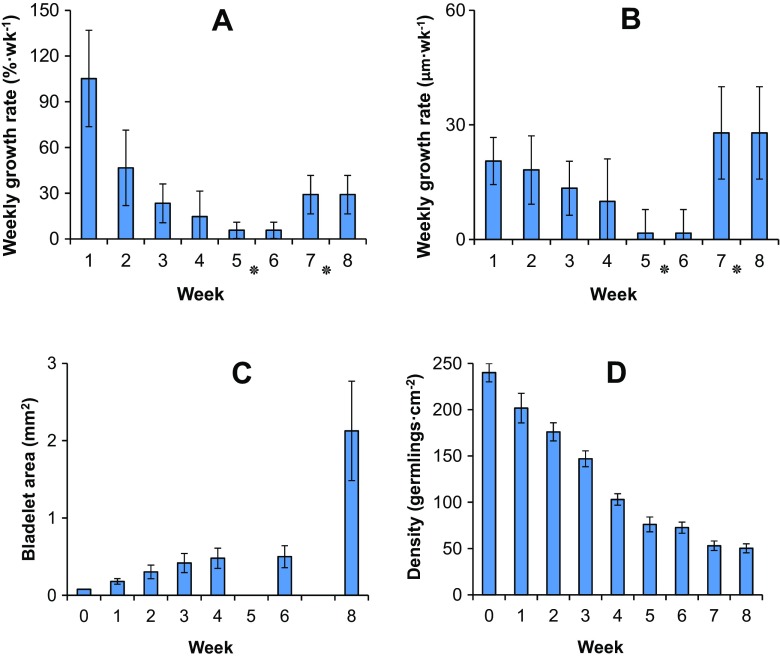
Growth characteristics of *Sargassum muticum* germlings over 8 weeks. The weekly growth rate as either **a** % wk^−1^, **b** μm wk^−1^ or **c** bladelet area. Also shown is **d** the decline in germling density due to detachment or death. Shown is mean ± standard deviation. * denotes week pairs where growth was derived from a single measurement

The growth rate was initially high in all conditions (105 ± 32% week^−1^), declining exponentially (*y* = 618.9e^−1.342x^, *R*^2^ = 0.93) to 6 ± 5% week^−1^ during weeks 5 and 6. Over the last 2 weeks, growth rate increased again to 17 ± 42% week^−1^ (Fig. 6a). Incremental growth was initially 21 ± 6 μm week^−1^, declined to only 2 ± 6 μm week^−1^ during weeks 5 and 6, then increased again to 28 ± 12 μm week^−1^ in weeks 7 and 8 (Fig. 6b).

The estimated frond area underwent a similar pattern, with area increasing from initially 0.08 to 0.5 mm^2^ in week 6 and then undergoing a rapid increase to 2.1 mm^2^ by week 8 (Fig. 6c). At settlement, the density was 240 ± 14 germlings cm^2^ (Fig. 6d). This declined linearly, at a rate of − 32.7 germlings cm^−2^ week^−1^ until week 5 (*r*^2^ = 0.98, *n* = 18). From weeks 5–8, this rate was only − 8.9 germlings cm^−2^ week^−1^. The density at week 8 was 53 ± 5 germlings cm^−2^, a 4.5-fold decrease from the settlement value.

### Effect of F/2-Si medium and GeO_2_

**Fig. 7:**
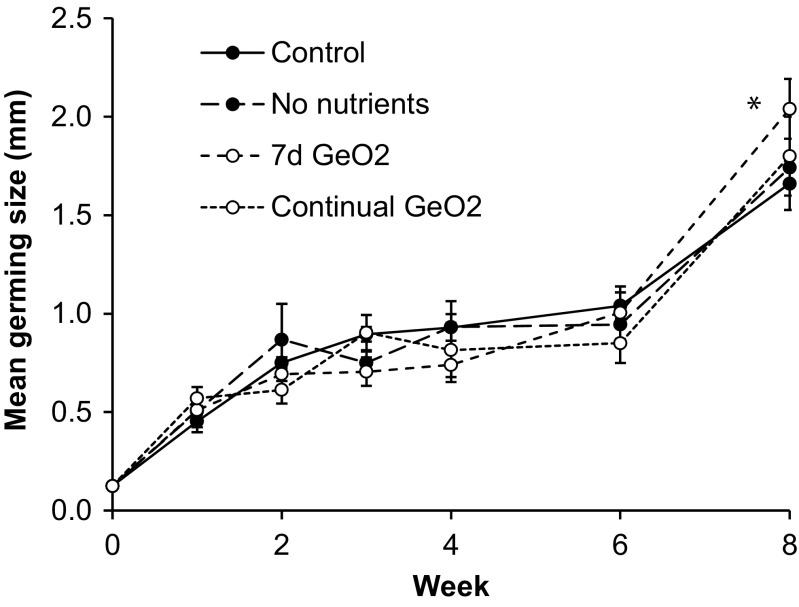
Mean germling size of over an 8-week experiment under four experimental conditions. Control: F/2-Si medium. No nutrients: seawater only. 7d GeO_2_: 0.125 mL of saturated GeO_2_ L^−1^ for the first 7 days of culture. Continual GeO_2_: GeO_2_ added throughout the experiment which was significantly different to the others at week 8 (*p < 0.005). Shown is the mean ± standard deviation

Nutrient supplementation was not necessary for the optimal growth of *S. muticum* germlings. The rate of growth of germlings cultured in either F/2-Si or only seawater was not significantly different (*p* > 0.05) and followed a near identical growth trajectory over the 8-week experiment (Fig. 7). Treatment with 0.125 mL L^−1^ of saturated GeO_2_ appeared to slightly depress growth over the first 4 weeks. By the end of the experiment, a continual exposure of GeO_2_ had not significantly affected growth (*p* > 0.05) compared to the control. On the other hand, after 8 weeks, the germlings exposed to GeO_2_ for only the first 7 days were significantly larger by 23% (AN: *F*_1,8=_17.1, *p* < 0.005; 2.04 ± 0.15 mm vs. 1.66 ± 0.14 mm in the control).

### Germling density and size of the six twines

**Fig. 8:**
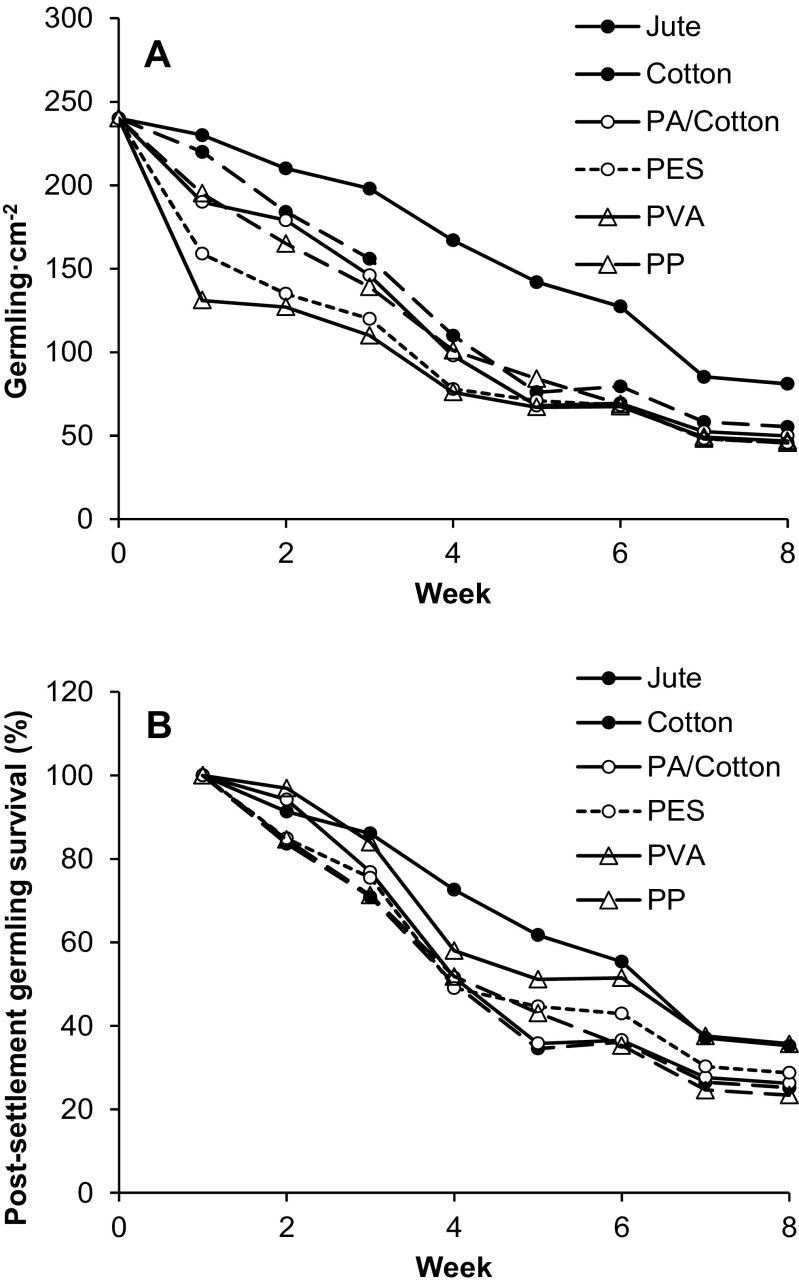
**a** Density of *S. muticum* germlings on six different twines over the course of the 8-week experiment. **b** % survival of germlings from weeks 1–8. Shown is mean, no error bars are shown for clarity

On all materials, germling density declined over the course of the experiment (Fig. 8a). Jute retained the highest density by the end of the experiment of 81 ± 16 germling cm^−2^, significantly different from all others (ANOVA: *F*_5,18_ = 16.5, *p* < 0.0001) which had 49 ± 4 germling cm^−2^ (*p* > 0.05). There did appear to be a difference in the initial retention of the germlings between the materials. The rougher, natural, materials, jute and cotton had the highest successful attachment after 1 week (92–96%). PA/cotton and PP had 79–81% whilst PVA and PES had the lowest at 55–66%. Following the first week, the density of surviving germlings declined fairly steadily on all materials (Fig. 8b). At week 8, the highest survival was seen on either jute or PVA (35–36%). Thereafter, these declined at 9.9 and 10.1 germling cm^−2^ week^−1^, respectively (LR, *r*^2^ = 0.93–0.98). On the other materials, 11.1% germling cm^−2^ were lost per week (LR, *r*^2^ = 0.93), resulting in a lower final survival at week 8 (23–29%).

**Fig. 9:**
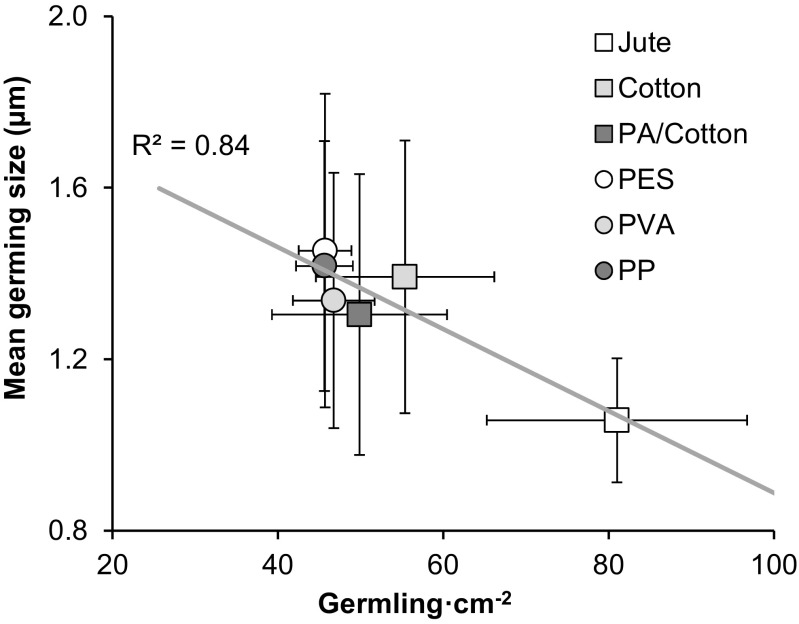
Density dependence between mean germling size of *S. muticum* at week 8 and the germling density across the six materials. Shown is the mean ± standard deviation

The final germling size on the different substrates varied, with lower growth seen on jute (1.06 ± 0.14) than other twines (1.38 ± 0.06). When the final density was considered, there was a clear negative density-dependent effect (*R*^2^ = 0.84). The largest germlings were seen at the lower densities, and the smaller germlings were found at the highest densities on jute (Fig. 9).

## Discussion

By manipulating the hatchery conditions used in the cultivation of a macroalga, the survival and development rate of juveniles can be maximised before outplanting. Resource efficiency can then be optimised by shortening the time needed and reducing wastage of juvenile material. In this study, we found that it took 8 weeks for the hatchery cultivation of *S. muticum* to yield germlings of 1.5–2 mm. At this size, they can be outplanted in the sea for cultivation as in related *Sargassum* spp. (Xie et al. 2013). The growth rate observed was similar to that observed in *S. thunbergii* (Zhao et al. 2008) but was slow when compared to many other cultivated Sargassaceae; 5 mm is reached in about a month by either *S. vachellianum* or *S. fulvellum* (Hwang et al. 2006; Chai et al. 2014). The growth rate was also low in comparison to other studies on *S. muticum* where 3–5 mm was achieved in a month post-settlement (Hales and Fletcher 1989; Steen 2004).

The reason for this slow growth was very likely due to the sub-optimal density used in this study. At high densities, intraspecific competition for resources such as light, CO_2_ or nutrients becomes intense. This resource limitation reduces the overall mean growth achieved by a population, leads to a large size inequity with the most successful individuals growing far larger than the majority of their compatriots. Also, self-thinning occurs as overgrowth leads to high mortality in the smaller individuals (Creed et al. 1998). When the density is low, each individual has sufficient resources, leading to fast growth, high survival and more similarly sized individuals across the whole population. This effect has been reported before in juvenile seaweeds (Reed et al. 1991; Steen and Scrosati 2004), including *S. muticum*, where it was found that germlings settled at 100 cm^−2^ had half the bladelet area of those settled at 10 germling cm^−2^ (Huggett et al. 2009). The initial density used in this study of 240 ± 10 germling cm^−2^ is far higher than the 20–65 germling cm^−2^ typically used in other *Sargassum* spp. experiments (McGuinness and Underwood 1986; McGuinness 1989; Hwang et al. 2006; Xie et al. 2013). This will have slowed the overall growth and increased mortality through intraspecific competition.

Very low densities will lead to even faster growth, as demonstrated in Steen (2003), where *S. muticum* cultured at 10 petridish^−1^ reached 10 mm after 36 days. However, within a hatchery, very low densities will lead to open substratum available for the colonisation of contaminating organisms such as other macroalgae. These will then cause interspecific competition for resources during the early development of the germlings. Therefore, it has been recommended that about 20 germling cm^−2^ is the minimum density for commercial production of the Sargassaceae (McGuinness 1989; Lüning and Pang 2003).

The developmental stages of *S. muticum* over the 8-week experiment agree with a previous description of germling growth given by Fletcher and Fletcher (1975) and are similar to those seen in other members of the Sargassaceae (Zhao et al. 2008; Xie et al. 2013). The rate at which germling length increased, slowed over the first 6 weeks from an incremental growth rate of 21 to only 2 μm week^−1^. This appears to be due to the switch in the development from growing in frond length to the development of basal buds and lateral lamina, by week 6 and rhizoid holdfast expansion by week 8. This spreading development was not captured by the weekly growth rate statistics, which was only based on germling length, leading to us underestimating the growth rate as these features developed. During weeks 6–8, the incremental growth rate and estimated bladelet area were seen to rapidly increase as length extension begins to accelerate again.

The first aim of this study was to determine whether supplementation of the growth medium with either nutrients or GeO_2_ could speed up the time taken for *S. muticum* germlings to reach an outplantable size. In many studies, nutrient media are enriched by KNO_3_ and KH_2_PO_4_ to concentrations of 10 and 1 mg L^−1^, respectively (McGuinness 1989). We tested F/2-Si medium which is known to be very suitable for the growth of many algae. The Tyndallised seawater we used contained low concentrations of inorganic nutrients at this time of year, 2.3 ± 0.3 μM nitrate and 0.2 ± 0.0 μM phosphate. Despite the enrichment of inorganic N and P by 8.2 and 0.36 mM, respectively, no benefit was seen to the growth size or development rate, and so additional nutrients were not necessary over the first 8 weeks. This appeared sufficient for the first 8 weeks of *S. muticum* cultivation. It may be that *S. muticum* germlings contain sufficient nutrient stores to fuel growth over this initial period allowing its development to be maintained regardless of the environmental nutrient concentration. This would be advantageous for an opportunistic space-grabbing species such as this. This contrasts with the results of Steen (2003), where germling growth was found to be significantly stimulated by nutrient enrichment compared to autoclaved seawater with similarly low concentrations of nutrients to this study. This discrepancy may be due to a difference in the initial nutrient quotient of *S. muticum* eggs from the two populations, Scotland (this study) or Norway (Huggett et al. 2009).

The overgrowth of juvenile macroalgae by fast-growing benthic diatoms can lead to complete elimination, patchy development or slowed growth (Kerrison, P, unpublished results). GeO_2_ is known to interfere with the formation of the diatom’s frustule causing growth inhibition (Lewin 1966) but can also inhibit phaeophyte macroalgae at higher concentrations (Markham and Hagmeier 1982). *Fucus spiralis* was found to suffer some growth inhibition at only 0.2 mg L^−1^, equivalent to only 0.04 mL of saturated GeO_2_ solution per L. Concentrations of 0.1–0.5 mL of saturated GeO_2_ solution per L have been shown to be effective to improve development in the kelp *S. latissima*; however, higher concentrations inhibit its growth (Shea and Chopin 2007). Supplementation of the *S. muticum* medium with 0.125 mL L^−1^ over the first week of cultivation resulted in 23% larger germlings, whereas continual treatment over 8 weeks gave no benefit. This is a similar treatment regime to that found optimal for the hatchery phase of *S. latissima* (Kerrison et al. 2016b). Therefore, this treatment appears suitable for a wide range of phaeophyte macroalgae.

In the second aim, six materials were tested for their suitability as growth substrata for *S. muticum* germlings. After 8 weeks, all materials, both natural and synthetic, were successfully colonised by the developing germlings and so are suitable substrates for the hatchery cultivation of *S. muticum*. Germlings on all materials displayed very similar survival over weeks 1–8 (~ 90% week^−1^) indicating that the attachment tenacity was similar, although very slightly higher on jute and PVA. It appears that the adhesive and spreading rhizoids of *S. muticum* germlings are very effective at attaching to various substrata. This agrees with its invasive ecology as a successful opportunistic space-grabber, able to colonise any available hard surface (Fletcher and Fletcher 1975; Norton 1977; Denny 1988).

PVA and PES twines had the highest week 1 losses of 34–55% of germlings, indicating that a weaker initial attachment was formed. In contrast, both jute and cotton only lost 4–8% of the settled germlings during this time. It is thought that the rougher surface of these natural materials was beneficial to the initial attachment, through physical entanglement of the germlings in the fibres. This agrees with previous reports that generally, rugose surfaces are more favourable for settlement and attachment. Increasing the surface area for attachment, allowing physical interlocking and providing micro-environments that shield juveniles from high flow that can cause detachment (Malm et al. 2003; Morrison et al. 2009; Long et al. 2010). On the smoother synthetic fibres, entanglement would be less common reducing successful settlement.

On nearly all materials, by week 8, the germling density had decreased to 49 ± 4 germling cm^−2^. Self-thinning of the population is most likely to be responsible (Creed et al. 1998) as the most successful individuals dominate and suppress the growth of smaller individuals (Steen and Scrosati 2004; Huggett et al. 2009). On jute, the final density was higher (81 ± 4 germling cm^−2^); however, the germlings were smaller and had more limited rhizoid development (data not shown). These characteristics are similar to those seen at week 6 on all other materials, indicating that the higher germling density retained by jute led to more intensive intraspecific competition and a slower overall development rate. Therefore, whilst it may initially appear, the jute is less suitable as a substrate due to slower growth, this is an artefact of higher germling density.

## Conclusions

Over an 8-week study on the cultivation of *S. muticum* germlings, we found that it was beneficial to add 0.125 mL L^−1^ of saturated GeO_2_ during the first week to inhibit the growth of diatom competitors. However, this benefit was lost if GeO_2_ was always added. Adding nutrients in the form of F/2-Si did not give any benefit to growth indicating that the zygotes carried sufficient nutrient reserves to reach ~ 2 mm. If larger germlings are required, nutrient dosing may be necessary once they are beyond this size. It was also determined that various twines are suitable for the attachment and cultivation of *S. muticum*, with rougher natural materials leading to higher initial settlement success. Jute retained the highest density of germlings at settlement making it highly suitable for cultivation. However, care must be taken to control the initial settlement density. In this study, higher germling densities lead to slower growth due to intraspecific competition. An initial density of ~ 20 germling cm^−2^ is recommended as is used for other members of the Sargassaceae.
